# Polyarylene Ether Nitrile/Modified Hollow Silica Composite Films for Ultralow Dielectric Properties and Enhanced Thermal Resistance

**DOI:** 10.3390/polym17121623

**Published:** 2025-06-11

**Authors:** Shuning Liu, Jinqi Wu, Yani Chen, Ting Zhang, Lifen Tong, Xiaobo Liu

**Affiliations:** School of Materials and Energy, University of Electronic Science and Technology of China, Chengdu 611731, China; liushuning@uestc.edu.cn (S.L.); 202321030422@std.uestc.edu.cn (J.W.);

**Keywords:** polyarylene ether nitrile, hollow silica microspheres, low-dielectric, high thermal resistance

## Abstract

Highly heat-resistant and low-dielectric materials are crucial for achieving high-frequency communication, high-density integration, and high-temperature stability in modern electronics. In this work, surface modification of hollow silica microspheres (HGMs) using a silane coupling agent ((3-aminopropyl)triethoxysilane, KH550) yielded KHGM particles with a coating content of approximately 9.3 wt%, which were subsequently incorporated into high-performance polyarylene ether nitrile (PEN) polymers to fabricate composite films. The modified nanoparticles demonstrated significantly enhanced compatibility with the polymer matrix, while their hollow structure effectively reduced the dielectric constant of the composite film. When loaded with 50 wt% KHGM particles, the PEN-based composite film exhibited an elevated glass transition temperature of 198 °C and achieved a dielectric constant as low as 2.32 at 1 MHz frequency, coupled with dielectric loss below 0.016; compared with pure PEN, the dielectric constant of PEN/KHGM-50% decreased by 26.47%. Additionally, the composite demonstrated excellent water repellency. These advancements provide high-performance material support for applications in electronic communications, aerospace, and related fields.

## 1. Introduction

In 5G communication systems, the rapid transmission of high-frequency signals imposes imperative demands on the dielectric properties of circuit boards and packaging materials [1,2,3,4]. Materials with low dielectric constants prove essential for minimizing signal crosstalk while ensuring stable and reliable communication performance [5,6,7]. Meanwhile, the ongoing trends toward miniaturization, integration, and higher operational speeds in electronic devices have substantially increased component density, leading to significant heat accumulation per unit area [8,9,10,11]. Without adequate thermal resistance, materials operating under elevated temperatures risk softening, deformation, or even thermal degradation [12,13,14]. Such failures could trigger critical failures such as circuit shorting and performance degradation, particularly in high-power or prolonged operational scenarios [15,16,17,18]. These challenges highlight the dual necessity for advanced materials that simultaneously optimize dielectric characteristics and thermal stability in next-generation electronics [19,20,21].

Polyarylene ether nitrile (PEN) is an aromatic polymer featuring a backbone rich in benzene rings and ether linkages, along with abundant cyanide groups on its side chains [22,23,24]. The high concentration of benzene rings in PEN’s main chain endows it with an elevated glass transition temperature (*T_g_*), exceptional mechanical properties, excellent heat resistance, and flame retardancy, making it irreplaceable in high-end applications such as electronics, aerospace, and precision machinery [25,26,27]. However, the strong polarity induced by the cyanide groups on PEN’s side chains necessitates strategies to further reduce its dielectric constant [28]. This is typically achieved through molecular structure design or compounding with low-dielectric fillers [29]. For instance, Tong et al. [30] synthesized a series of phthalonitrile-terminated PEN (PEN-t-Ph), where cross-linking reactions between terminal phthalonitrile groups formed phthalocyanine rings. This modification not only enhanced the polymer’s *T_g_* but also reduced its dielectric constant. Peng et al. [31] introduced MOF-3 into carboxyl-containing PEN, resulting in a PEN-M-3 composite film with an ultralow dielectric constant of 2.33 at 1 MHz. Additionally, Zhang et al. [32] incorporated octaphenyl polyhedral oligomeric silsesquioxane (OPOSS) into PEN and developed a dual cross-linked network via thermal treatment, simultaneously improving the polymer’s *T_g_* and lowering its dielectric constant.

This study developed a composite film by incorporating surface-modified HGMs into bisphenol AP-type PEN. The HGM particles were functionalized with KH550 to yield KHGM particles. The KH550 modification significantly enhanced the interfacial compatibility and dispersion uniformity of HGMs within the PEN matrix, while the hollow architecture of HGMs effectively reduced the dielectric constant of the composite. Systematic investigations were conducted to evaluate the impact of varying KHGM loading ratios on the thermal, dielectric, and surface properties of the films. The optimized composite film demonstrated exceptional thermal stability with a glass transition temperature, ultralow dielectric characteristics, and notable hydrophobicity. These synergistic improvements highlight the material’s potential for advanced applications in high-frequency electronics and extreme thermal environments.

## 2. Experimental

### 2.1. Materials

Bisphenol AP and 2,6-dichlorobenzonitrile (DCBN, 96%) were purchased from Shanghai Titan Technology Co., Ltd., Shanghai, China. Potassium carbonate (K_2_CO_3_, 98%), toluene, N-methyl pyrrolidone (NMP), NaOH (99%), and hydrochloric acid (HCl, 37%) were supplied by Tianjin BODI Chemicals, Tianjin, China. HGMs were obtained from Ningbo YUMU Chemicals, Ningbo, China. All purchased reagents were commercially available and used without further purification.

### 2.2. Preparation of PEN

In a 250 mL three-necked flask equipped with a thermometer, Dean-Stark trap, reflux condenser, and mechanical stirrer, bisphenol AP (0.12 mol), DCBN (0.12 mol), K_2_CO_3_ (0.17 mol), 75 mL of NMP, and 25 mL of toluene were added. The mixture was mechanically stirred and heated to 150 °C for azeotropic dehydration over 3 h to facilitate the reaction. Upon further heating to 180 °C, the polymerization proceeded until a distinct threading phenomenon was observed. The resultant product was precipitated into cold water to yield crude BPA-PEN. The crude product was pulverized, purified sequentially with diluted hydrochloric acid and boiling water to remove residual KCl and unreacted monomers, and finally dried under vacuum at 100 °C for 24 h. The chemical structure of the synthesized BPA-PEN is illustrated in Figure S1 from the Supplementary Materials.

### 2.3. Synthesis of KHGM

**Figure 1:**
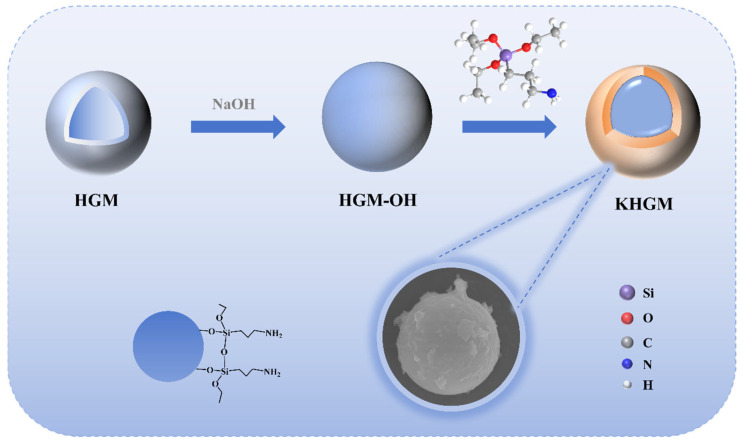
Schematic illustration of the KHGM preparation process.

A total of 12 g of HGMs were dispersed in 300 mL of 2% NaOH solution and refluxed with stirring at 80 °C in an oil bath for 2 h. The mixture was filtered and thoroughly washed with deionized water until a neutral pH was achieved, followed by drying in an oven for 12 h to obtain hydroxyl-functionalized HGMs (HGM-OH). Next, 300 mL of an ethanol/water mixture (3:1 *v*/*v*) was added to a 500 mL three-necked flask, followed by the addition of 1.5 g of KH550. The solution was adjusted to pH 4–5 using acetic acid and stirred at room temperature for 1 h [33]. Subsequently, 5 g of HGM-OH was introduced into the mixture, and the reaction was refluxed with vigorous stirring at 80 °C for 3 h. The resulting powder was collected by filtration, washed repeatedly with ethanol and deionized water, and dried in an oven for 12 h to yield KHGMs. The preparation flowchart of KHGMs is shown in Figure 1.

### 2.4. Preparation of PEN/KHGM Films

PEN/KHGM composite films were prepared using a solution casting method. Specifically, 2 g of PEN was dissolved in 10 mL of N-methyl-2-pyrrolidone (NMP), followed by the addition of KHGM powder at mass fractions of 10 wt%, 20 wt%, 30 wt%, 40 wt%, and 50 wt%. The mixtures were ultrasonicated for 30 min and mechanically stirred to ensure homogeneity. The dispersed solutions were then cast onto glass substrates (25 cm × 25 cm) and subjected to a stepwise heating protocol: 80 °C (1 h) → 100 °C (1 h) → 120 °C (1 h) → 160 °C (2 h) → 200 °C (2 h). The resulting films were designated as PEN/KHGM-10%, PEN/KHGM-20%, PEN/KHGM-30%, PEN/KHGM-40%, and PEN/KHGM-50%, respectively. For comparison, PEN/HGM films were fabricated using an identical procedure.

### 2.5. Characterization

This work employed a JSM-5900LV scanning electron microscope (SEM) from JEOL Ltd. (Japan, Tokyo) to investigate the microstructure of both HGMs (hollow glass microspheres) and KHGMs (modified HGMs). FTIR measurements were conducted using a Shimadzu 8400S FTIR spectrometer (Japan, Tokyo) to determine the structure of KHGMs. Thermogravimetric analysis (TGA) was performed using a TA Instruments Q50 under a nitrogen atmosphere to confirm the thickness of the silane coupling agent layer on KHGMs from TA Instruments (New Castle, DE, USA). Differential Scanning Calorimetry (DSC) analysis of the PEN composite films was carried out using a TA Instruments DSC Q100. The temperature was ramped from 25 °C to 350 °C at a heating rate of 10 °C/min. The rheological properties of the composite films were measured using a rotational rheometer (TA Instruments, AR-G2). The dielectric properties of the PEN dielectric composites were determined using a TH 2819A Precision LCR Meter from Tong Hui Ltd. (Changzhou, China).

## 3. Results and Discussion

**Figure 2:**
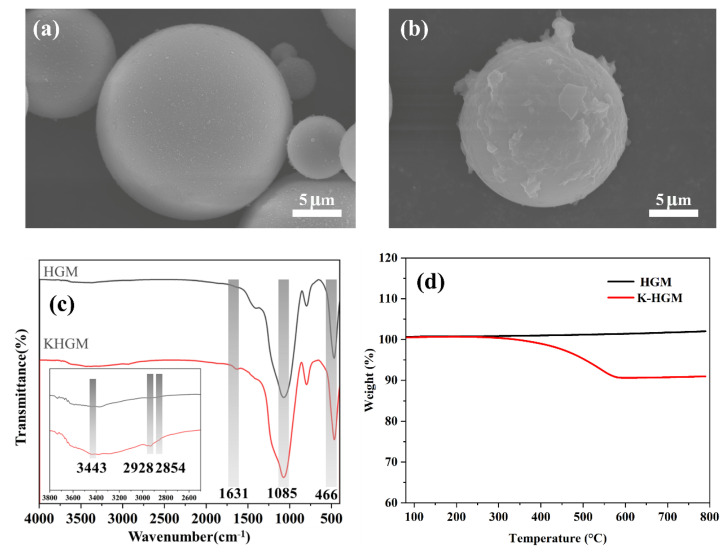
(**a**) SEM image of an HGM. (**b**) SEM image of a KHGM. (**c**) FTIR of a KHGM and HGM. (**d**) TGA of an HGM and KHGM.

To confirm the successful synthesis of KHGMs, hollow silica microspheres (HGMs) and KH550-modified HGMs (KHGMs) were characterized using scanning electron microscopy (SEM), Fourier-transform infrared spectroscopy (FTIR), and thermogravimetric analysis (TGA). Figure 2a,b present SEM images of HGMs and KHGMs, respectively. The KHGM surface exhibits an abundance of coated structures with a rougher yet relatively uniform morphology compared to pristine HGMs, confirming successful surface modification. Notably, HGMs maintained structural integrity with minimal fractures (Figure S2), and their average particle size was measured as 7.47 μm (data summarized in Figure S3). FTIR spectra of HGMs and KHGMs are shown in Figure 2c. Both materials display strong absorption peaks at 1085 cm^−1^ and 466 cm^−1^, attributed to the stretching vibrations of Si-O-Si bonds. Distinctive new peaks emerge in KHGMs at 2928 cm^−1^ and 2854 cm^−1^, corresponding to the asymmetric and symmetric stretching vibrations of methyl and methylene groups in the grafted silane coupling agent. Additionally, the enhanced absorption peaks at 3443 cm^−1^ (O-H stretching) and 1631 cm^−1^ (O-H bending) further validate the successful functionalization of KHGMs [34]. TGA curves in Figure 2d reveal thermal decomposition behavior. The weight loss of KHGMs at elevated temperatures corresponds to the degradation of KH550, with a calculated coating amount of approximately 9.3 wt% on the modified HGM surface.

**Figure 3:**
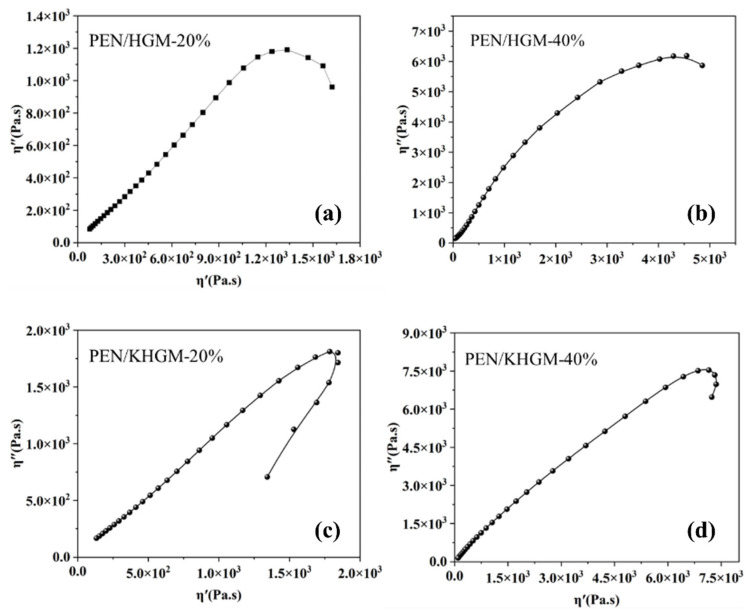
Cole-Cole plot curves of (**a**) PEN/HGM-20%, (**b**) PEN/HGM-40%, (**c**) PEN/KHGM-20%, and (**d**) PEN/KHGM-40%.

Due to the strong tendency of HGMs to agglomerate within polymer systems, this study employed KH550 to enhance the compatibility between HGMs and the PEN matrix. The viscoelastic behavior of the multicomposite system was analyzed to evaluate the impact of fillers on polymer relaxation dynamics [35,36]. Figure S4 shows the viscosity curves of the PEN composite films as a function of frequency. From the viscosity curves of the PEN composite films, it can be observed that at low frequencies, the viscosity exhibits a distinct relaxation process. This behavior is attributed to the rigidity of the PEN molecular chains, intermolecular interactions, and the formation of chain entanglements. Furthermore, a pronounced shear-thinning behavior is observed when the shear frequency exceeds 0.2 Hz. Additionally, while the PEN/HGM and PEN/KHGM composite materials display a similar viscosity profile, the viscosity of the system is further increased due to the friction between the PEN matrix and the KHGM surface, as well as the physical interlocking effects formed. Figure 3 presents Cole-Cole plots for PEN/HGM-20%, PEN/HGM-40%, PEN/KHGM-20%, and PEN/KHGM-40%. The arc shape and the size of the arc radius of the Cole-Cole curve can reflect the interface compatibility and the changes in interaction. As shown in Figure 3c, the Cole-Cole curve of PEN/KHGM-20% approximates a semicircular arc. However, increasing the filler content induces significant deviation from semicircular profiles. Such deviations reflect variations in the interfacial compatibility between the fillers and the PEN matrix. Notably, the flattened tails of the Cole-Cole curves for PEN/HGM-20% and PEN/HGM-40% at the low-frequency region suggest prolonged relaxation times attributed to poor filler-matrix compatibility. Similar trends were observed in composites with other filler loadings in Figure S5. These results confirm that the KH550 modification optimizes the molecular chain mobility of PEN and improves interfacial compatibility compared to unmodified HGMs, thereby promoting uniform dispersion of nanoparticles in the composite.

**Figure 4:**
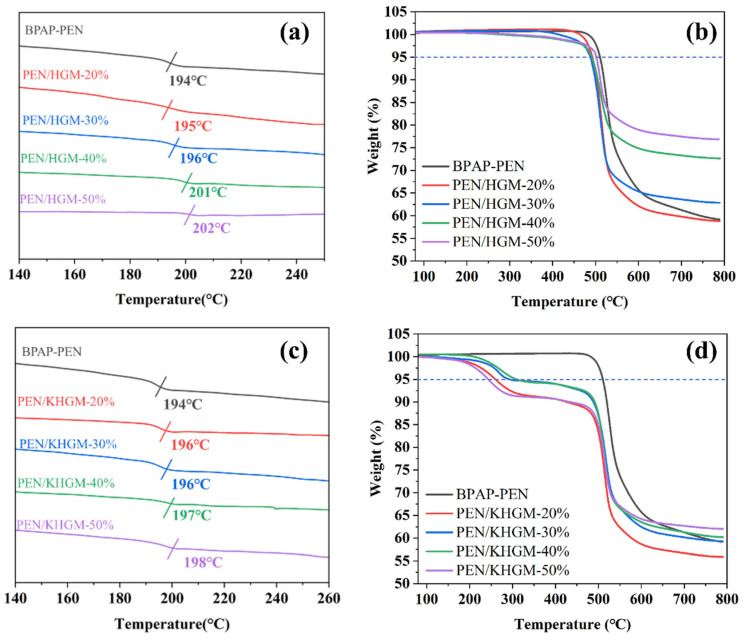
DSC curves of (**a**) PEN and PEN/HGM composites, (**c**) PEN and PEN/KHGM composites; TGA curves of (**b**) PEN and PEN/HGM composites, (**d**) PEN and PEN/KHGM composites.

Low-dielectric films with excellent heat resistance and thermal stability are essential for ensuring the high performance, reliability, and longevity of electronic devices. The copolymer exhibits outstanding thermal stability, enabling reliable operation under extreme temperatures. Figure 4a,c present the DSC curves of PEN composites, revealing high *T_g_* across all samples. Notably, the *T_g_* increases with higher filler content due to the restricted mobility of PEN molecular chains caused by the dispersion of rigid particles. At 50 wt% KHGM loading, the *T_g_* of PEN/KHGM-50% reaches 198 °C, demonstrating enhanced chain immobilization at elevated filler concentrations. Figure 4b,d display the TGA curves of the composites. Despite the relatively lower thermal decomposition temperature of KH550 in KHGMs compared to the PEN matrix, the *T*_5%_ (temperature at 5% weight loss) of PEN/KHGM-50% remains above 240 °C even at maximum filler loading. This value significantly exceeds its *T_g_*, underscoring the material’s robustness for high-temperature applications.

**Figure 5:**
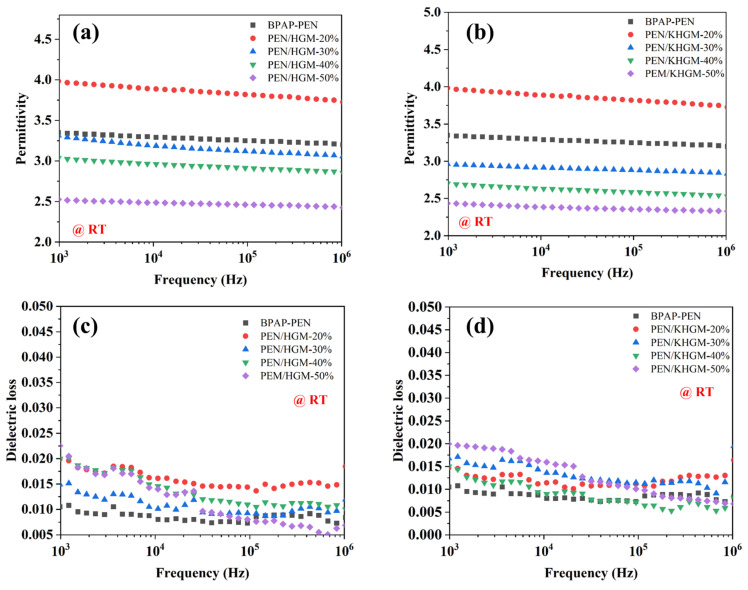
Dielectric constant of (**a**) PEN and PEN/HGM composites, (**b**) PEN and PEN/KHGM composites; dielectric loss of (**c**) PEN and PEN/HGM composites, (**d**) PEN and PEN/KHGM composites.

Dielectric properties are critical in designing advanced materials for high-frequency communication. Figure 5 illustrates the frequency-dependent variations in dielectric constant (ε) and dielectric loss (tanδ) of the composites at room temperature. As the applied electric field frequency increases, a slight decline in ε is observed, attributed to dipole polarization relaxation. Notably, the dielectric constant of PEN/KHGM composites progressively decreases with higher KHGM loading in Figure 5b, reaching a minimum ε of 2.32 at 1 MHz for PEN/KHGM-50%; compared with pure PEN, the dielectric constant of PEN/KHGM-50% decreased by 26.47%. This reduction is primarily due to the hollow structure of KHGMs, which introduces air voids into the PEN matrix, effectively lowering the overall dielectric constant. Furthermore, compared to PEN/HGM composites at equivalent filler loadings, as shown in Figure 5a, PEN/KHGM exhibits significantly lower ε values. This improvement stems from the enhanced dispersion of KHGMs within the polymer matrix via surface modification, which mitigates particle agglomeration and reduces interfacial polarization effects. The variation in the dielectric properties of the PEN composite films aligns with their viscoelastic behavior. Compared to HGMs, the microstructure on the KHGM surface enhances the interfacial interaction with the composite by forming physical interlocking effects with the PEN resin matrix. This improves the interfacial compatibility, enabling better dispersion of KHGMs within the polymer matrix. Consequently, this alleviates particle agglomeration and reduces the interfacial polarization effect. Therefore, the PEN/KHGM composite exhibits higher viscosity and a lower dielectric constant. Furthermore, the dielectric loss behavior of the composites is shown in Figure 5c,d. Owing to superior compatibility between KHGMs and PEN, interfacial polarization-induced losses are minimized, and KHGMs demonstrate more uniform dispersion than unmodified HGMs. Consequently, PEN/KHGM composites exhibit markedly lower tanδ values. Even at 50 wt% KHGM loading, PEN/KHGM-50% maintains a tanδ below 0.02, meeting the stringent requirements for low-loss dielectric materials in high-frequency applications. The temperature-dependent dielectric measurements of PEN/KHGM-40% are plotted in Figure S6. From the figure, it is readily apparent that once the test temperature exceeds the *T_g_* of the polymer material, the intensified molecular motion of the polymer chains leads to a sharp increase in polarization capability, resulting in a corresponding sharp rise in both the dielectric constant and loss. Conversely, when the test temperature falls below the *T_g_* of the polymer composite film material, both the dielectric constant and dielectric loss remain remarkably stable. Therefore, the PEN composite film dielectric can be reliably used at temperatures below its *T_g_*, demonstrating excellent thermal stability.

**Figure 6:**
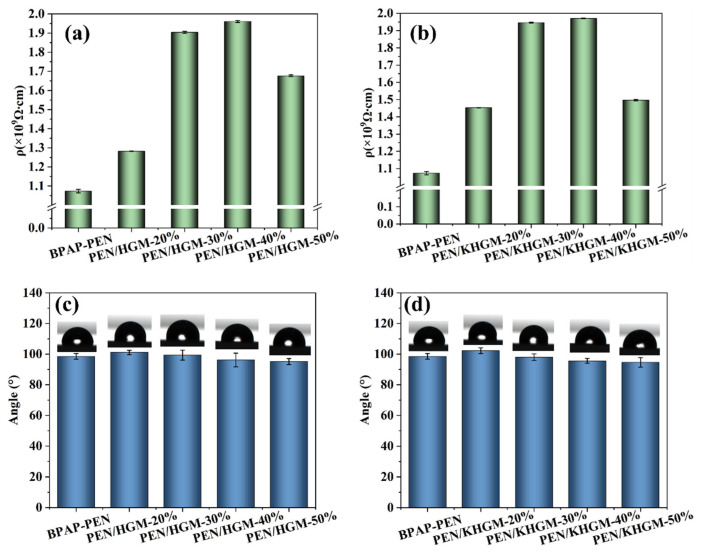
Volume resistivity of (**a**) PEN and PEN/HGM composites, (**b**) PEN and PEN/KHGM composites; contact angle of (**c**) PEN and PEN/HGM composites, (**d**) PEN and PEN/KHGM composites.

The volume resistivity of PEN/HGM and PEN/KHGM composite films is shown in Figure 6a,b. The resistivity increases with higher hollow filler content, attributed to the enhanced insulating properties of the composites. Notably, PEN/KHGM-40% achieves a maximum volume resistivity of 1.97 × 10^9^ Ω·cm, reflecting superior electrical insulation due to the improved dispersion of KHGMs. In the context of device miniaturization and high-frequency operation, surface hydrophobicity becomes critical for low-dielectric materials. Hydrophilic surfaces can degrade dielectric performance, induce signal delays, and compromise device reliability. As illustrated in Figure 6c,d, all PEN composite films exhibit water contact angles exceeding 90°, confirming their robust hydrophobicity. This property ensures stable dielectric performance in humid environments. Furthermore, the hydrophobic surface inhibits contaminant adhesion and minimizes the formation of conductive pathways, effectively preventing short-circuit failures. These characteristics collectively extend the film’s service life and enhance operational stability in advanced electronic systems.

## 4. Conclusions

This study demonstrates the successful fabrication of high-performance PEN-based composite films by incorporating surface-modified KHGMs via KH550 silane coupling agent functionalization. The synergistic interplay between the optimized interfacial compatibility (achieved through silane modification) and the intrinsic hollow structure of KHGMs endows the composites with exceptional thermal stability, ultralow dielectric properties, and robust hydrophobicity. The KH550 modification significantly enhances the dispersion uniformity of KHGMs within the PEN matrix, suppressing filler agglomeration and reducing interfacial polarization, which directly contributes to a dielectric constant as low as 2.32 (at 1 MHz) and a dielectric loss below 0.02 even at 50 wt% filler loading. Simultaneously, the composites exhibit a high *T_g_* at 198 °C and thermal decomposition stability (*T*_5%_ > 240 °C), ensuring reliability in extreme thermal environments. Furthermore, the films achieve remarkable electrical insulation and water contact angles, effectively mitigating moisture absorption, surface contamination, and electrical leakage risks. These integrated properties address critical challenges in high-frequency communication, 5G systems, and aerospace applications, where signal integrity, thermal management, and environmental durability are paramount. This work not only advances the design of multifunctional dielectric materials but also provides a scalable interfacial engineering strategy for next-generation polymer composites.

## Data Availability

The original contributions presented in this study are included in the article. Further inquiries can be directed to the corresponding authors.

## Supplementary Materials

The following supporting information can be downloaded at: https://www.mdpi.com/article/10.3390/polym17121623/s1, Figure S1: Synthesis route of BPAP-PEN; Figure S2: SEM image of broken HGM; Figure S3: Average particle size of HGM; Figure S4: Average particle size of HGM; Figure S5: Cole-Cole plot curves of (a) PEN/HGM-30%, (b) PEN/HGM-50%, (c) PEN/KHGM-30% and (d) PEN/KHGM-50%; Figure S6: (a) Permittivity and (b) dielectric loss of temperature of PEN/KHGM-40%.
