# Supplementary material for: Polyarylene Ether Nitrile/Modified Hollow Silica Composite Films for Ultralow Dielectric Properties and Enhanced Thermal Resistance
Source: Polymers (Basel). 2025 Jun 11;17(12):1623. doi: 10.3390/polym17121623 (PMC12197190; doi:10.3390/polym17121623)
Supplement: Supplementary file 1 [file polymers-17-01623-s001.zip › polymers-3674187-supplementary.pdf]

Supporting information

Polyarylene Ether Nitrile/Modified Hollow Silica Composite Films for Ultralow Dielectric Properties and Enhanced Thermal Resistance

Shuning Liu, Jinqi Wu, Yani Chen, Ting Zhang, Lifen Tong * and Xiaobo Liu *

School of Materials and Energy, University of Electronic Science and Technology of China,
Chengdu 611731, China; liushuning@uestc.edu.cn (S.L.);
202321030422@std.uestc.edu.cn (J.W.); 202321030401@std.uestc.edu.cn (Y.C.);
202311030527@std.uestc.edu.cn (T.Z.)
* Correspondence: tonglifen0214@uestc.edu.cn (L.T.); liuxb@uestc.edu.cn (X.L.)

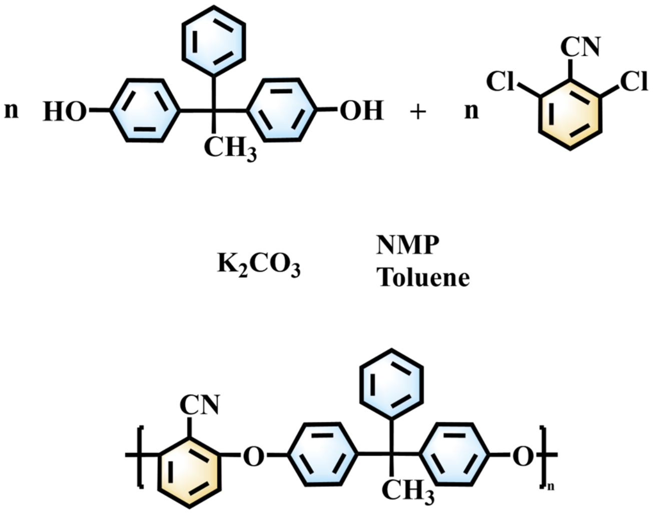

Figure S1. Synthesis route of BPAP-PEN.

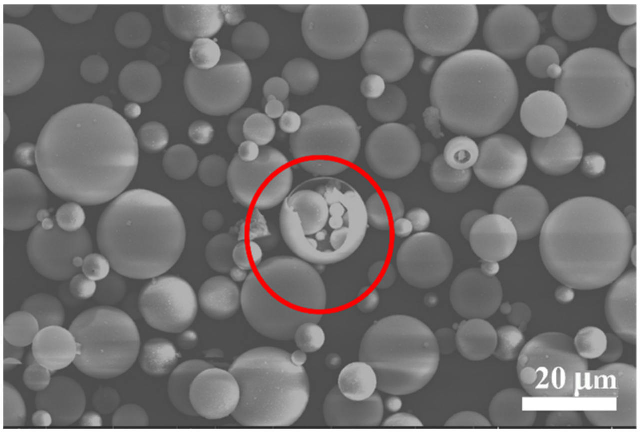

Figure S2. SEM image of broken HGM.

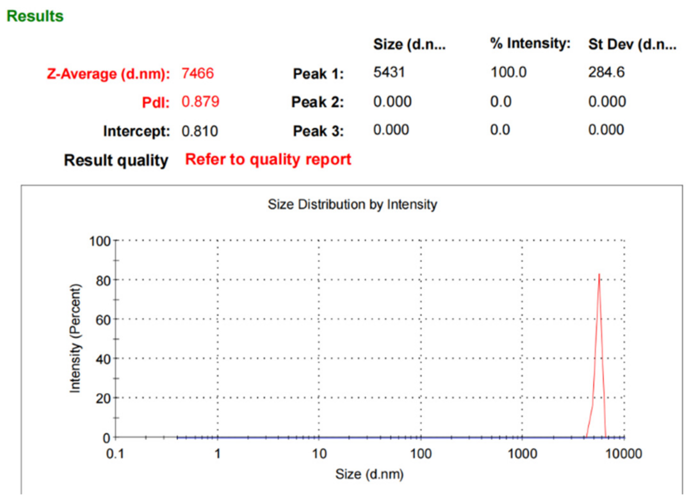

Figure S3. Average particle size of HGM.

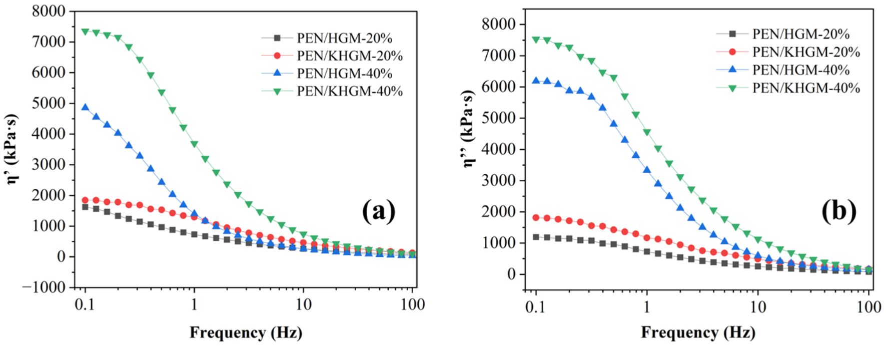

Figure S4. (a) Viscosity real part and (b)Viscosity imaginary part of PEN/HGM-20%, PEN/HGM-40%, PEN/KHGM-20% and PEN/KHGM-40%.

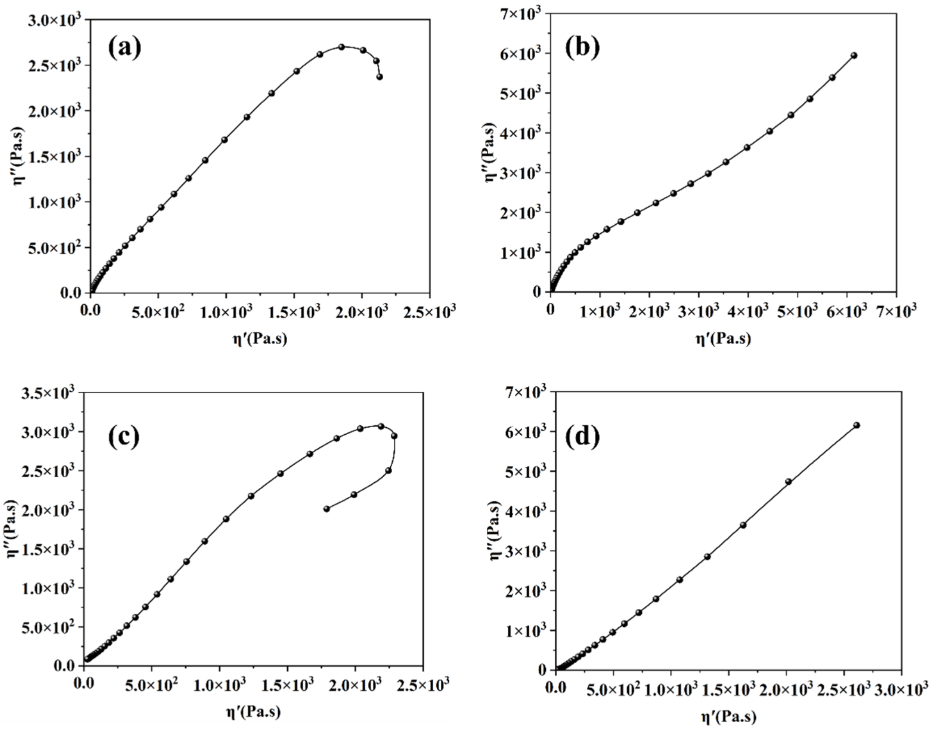

Figure S5. Cole-Cole plot curves of (a) PEN/HGM-30%, (b) PEN/HGM-50%, (c) PEN/KHGM-30%

and (d) PEN/KHGM-50%.

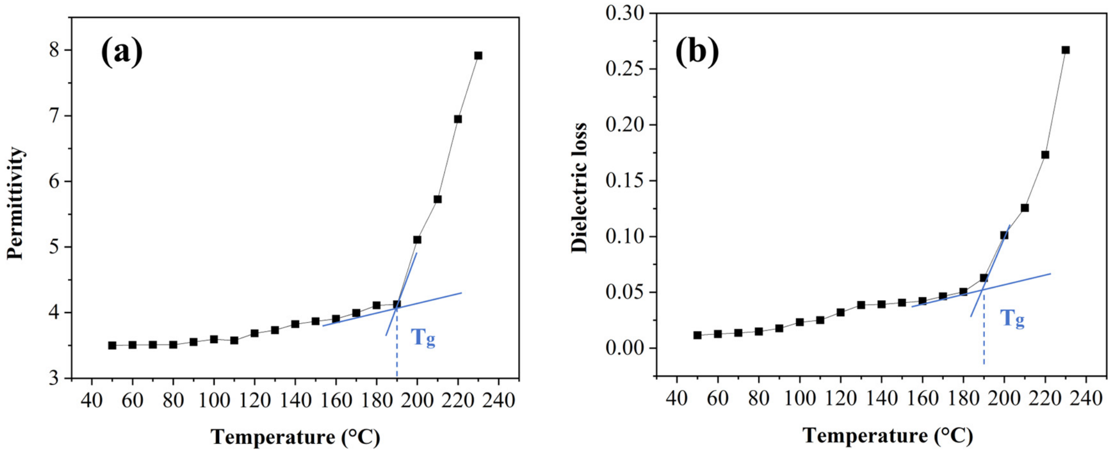

Figure S6. (a) Permittivity and (b) dielectric loss of temperature of PEN/KHGM-40%.
